# Liver biopsy-proven non-alcoholic fatty liver disease predicts no impact on antiviral response in patients with chronic hepatitis B

**DOI:** 10.1016/j.clinsp.2024.100493

**Published:** 2024-09-26

**Authors:** Miao-Yang Chen, Shun-Xin Li, Zhi-Xiang Du, Qing-Fang Xiong, Yan-Dan Zhong, Du-Xian Liu, Yong-Feng Yang

**Affiliations:** a Department of Infectious Disease and Liver Disease, The Second Hospital of Nanjing, Affiliated to Nanjing University of Chinese Medicine, Nanjing, Jiangsu, China; b Department of Pathology, The Second Hospital of Nanjing, Affiliated to Nanjing University of Chinese Medicine, Nanjing, Jiangsu, China

**Keywords:** Antiviral Response, Chronic hepatitis B, Non-alcoholic fatty liver disease, Non-alcoholic steatohepatitis

## Abstract

**Objective:**

The role of Non-Alcoholic Fatty Liver Disease (NAFLD) on antiviral response in Chronic Hepatitis B (CHB) remains unclear. Previous studies mainly focus on the impact of the Non-Alcoholic Fatty Liver (NAFL) on antiviral efficacy, whereas the role of Non-Alcoholic Steatohepatitis (NASH) has not been highlighted. The authors aimed to investigate the association of NAFLD (NAFL and NASH), viral and histological characteristics with antiviral response.

**Methods:**

The authors collected data of treatment-naïve CHB patients who underwent liver biopsy. All these patients received antiviral monotherapy and 48-week follow-up. The antiviral response was evaluated by Kaplan-Meier analysis. Cox regression analysis identified the variables associated with antiviral response.

**Results:**

Overall, 120 treatment-naïve CHB patients were enrolled, with 49.2 % (59/120) of them were complicated by NAFLD. Male (Odd Ratio [OR = 4.222], 95 % Confidence Interval [95 % CI 1.620-11.003]) and overweight (OR = 8.709, 95 % CI 3.355-22.606) were independent predictors for concurrent NAFLD. After 48-week follow-up, the authors found that the overall antiviral response did not differ between CHB patients with and without concomitant NAFL/NASH (p > 0.05). High viral load (Hazard Ratio [HR = 0.522], 95 % CI 0.286-0.952), advanced fibrosis (HR = 2.426, 95 % CI 1.256-4.686), and moderate-to-severe interface hepatitis (HR = 2.541, 95 % CI 1.406-4.592) were significantly correlated with antiviral response after 8-week follow-up.

**Conclusion:**

Neither NAFL nor NASH had an impact on antiviral therapy for CHB. It was low hepatitis B load, advanced fibrosis, and moderate-to-severe interface hepatitis that contributed to the virological response.

## Highlights

• NAFLD is increasingly common in patients with chronic hepatitis B.
• Neither NAFL nor NASH has an effect on the antiviral efficacy of chronic hepatitis B.
• Viral load and degree of inflammation and fibrosis influence antiviral response.

## Introduction

Chronic Hepatitis B (CHB) and Non-Alcoholic Fatty Liver Disease (NAFLD) are two common chronic liver diseases, with a prevalence of approximately 3.5 % and 30.0 %, respectively.^1^^,^^2^ The co-existence of CHB and NAFLD is encountered frequently in clinical practice, with a reported NAFLD in 13.6 % to 59.3 % of patients with CHB, indicating the significance of clarifying the interaction between the two diseases.^3^

Currently, extensive studies have explored the impact of concurrent NAFLD on CHB, and what can be established is that concurrent NAFLD might be a protective factor for CHB, as reflected by concurrent NAFLD as a predictor for Hepatitis B surface Antigen (HBsAg) seroclearance,^3^, ^4^, ^5^ the significantly earlier age of achieving spontaneous HBsAg seroclearance,^6^ and the negative correlation between NAFLD and Hepatitis B Virus (HBV) load.^7^, ^8^, ^9^ At the same time, however, concurrent NAFLD can aggravate fibrosis.^3^^,^^8^^,^^10^, ^11^, ^12^

In addition to these mentioned known effects, there is still no general agreement about the role of concurrent NAFLD on the antiviral efficacy of CHB. Previous clinical research has shown that CHB with hepatic steatosis had poor or no impact on Nucleos(t)ide Analogues (NAs) therapy.^10^^,^^13^, ^14^, ^15^, ^16^, ^17^, ^18^, ^19^ But based on the stage of disease progression, NAFLD broadly comprises two subtypes, the non-progressive form of NAFLD, Non-Alcoholic Fatty Liver (NAFL), and the progressive form of NAFLD, Non-Alcoholic Steatohepatitis (NASH).^20^ The above studies have merely focused on the effect of hepatic steatosis without distinguishing NASH from it histologically.^13^, ^14^, ^15^, ^16^, ^17^, ^18^, ^19^ Little attention has been paid to the role of NAFL and NASH on antiviral response, separately. Moreover, the studies assessed hepatic steatosis by abdominal ultrasound^13^^,^^15^, ^16^, ^17^, ^18^ or Vibration-Controlled Transient Elastography (VCTE),^14^ which was not the gold standard for diagnosis. Hence, the different stages of disease progression (NAFL or NASH) for NAFLD and diagnostic methods, likely explain the inconsistent conclusions. As outlined above, it is urgent to elucidate the role of NAFL/NASH on the antiviral response in chronic hepatitis B to clarify therapeutic implications.

Therefore, this study aimed to determine the incidence and independent risk factors for concurrent NAFLD among CHB patients who underwent liver biopsy and investigated the response of concurrent NAFL/NASH to antiviral therapy, which might contribute to a deeper understanding of the interplay between NAFLD and CHB. Moreover, the authors also ascertained the correlation between viral (Hepatitis B Virus Deoxyribonucleic Acid [HBV DNA] load and Hepatitis B e Antigen [HBeAg] status) and histological (degree of fibrosis and interface hepatitis) characteristics and antiviral response.

## Materials and methods

### Study population

**Fig. 1:**
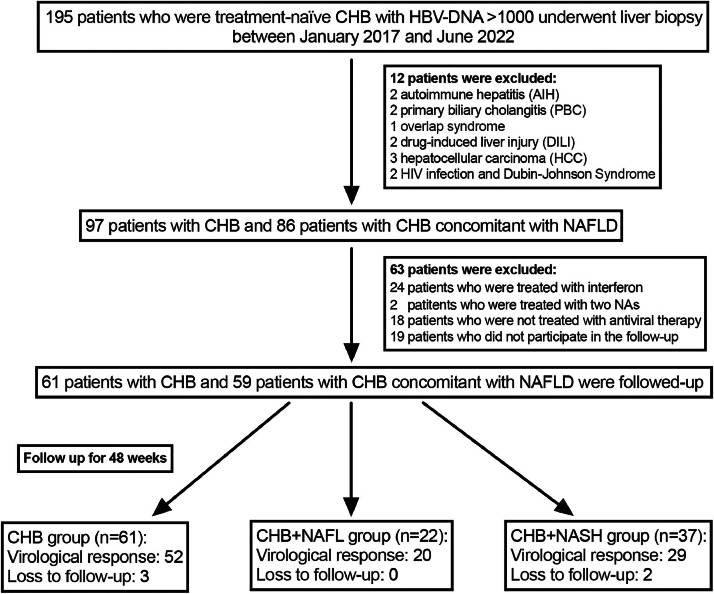
Flowchart of the study design. Abbreviations: CHB, chronic hepatitis B; HBV-DNA, hepatitis B virus deoxyribonucleic acid; NAFL, non-alcoholic fatty liver; NAFLD, non-alcoholic fatty liver disease; NAs, Nucleos(t)ide analogues; NASH, non-alcoholic steatohepatitis.

This retrospective study recruited treatment-naïve CHB with or without concomitant NAFLD who underwent liver biopsy in the Second Hospital of Nanjing, China, from January 2017 to June 2022. The inclusion criteria were as follows: (i) Serum positive for HBsAg ≥ 6 months; (ii) Serum HBV DNA load ≥ 3 log_10_ IU/mL; (iii) Patients who were not given antiviral therapy previously; (iv) Patients who underwent liver biopsy. The criteria for exclusion were as follows: (i) Presence of other chronic liver diseases, including Autoimmune Hepatitis (AIH), Primary Biliary Cholangitis (PBC), Overlap Syndrome (OS), Drug induced Liver Injury (DILI), Alcohol-Related Liver Diseases (ALD), and other viral infection; (ii) Patients who were diagnosed with Hepatocellular Carcinoma (HCC); (iii) Patients who lacked baseline and follow-up data; (iv) Patients who were treated with two NAs or with interferon, or were not receiving antiviral therapy. ALD was defined as the presence of excessive alcohol consumption (≥ 30 g per day for male and ≥ 20 g per day for female).^21^ All patients were followed for 48 weeks. Flowchart of the study design was shown in Fig. 1.

This study conformed to the ethical guidelines of the Declaration of Helsinki and was approved by the Ethics Committee of the Second Hospital of Nanjing (No. 2022-LY-kt101). The data were anonymous, and the requirement for informed consent was waived by the Medical Ethics Committee of the Second Hospital of Nanjing. As an observational study, this study follows the STROBE statement.

### Date collection

Demographic data (age and gender), anthropometric measurements, history of alcohol intake, biochemical indicators, and virological indicators were retrospectively collected. The data for anthropometric assessments included height, weight, and blood pressure. Biochemical indicators, including Total Bilirubin (TB), albumin, globulin, Alanine Aminotransferase (ALT), Aspartate aminotransferase (AST), γ-Glutamyl Transferase (γ-GT), Alkaline Phosphatase (ALP), and Fasting Blood Glucose (FBG), were tested with automatic biochemistry analyzer (Roche Cobas c701; Basel, Switzerland). HBV DNA was tested by a real-time Polymerase Chain Reaction (PCR) kit with a lower detection limit of 500 IU/mL (Sansure Biotech INC., Changsha, China), serum HBsAg and HBeAg were detected with an automated chemiluminescence analyzer (Roche Cobas e602; Basel, Switzerland). HBV DNA was categorized into 2 groups (low viral load group: HBV DNA < 6log_10_ IU/mL; high viral load group: HBV DNA ≥ 6 log_10_ IU/mL).

The metabolic-related risk was defined as the presence of at least one of the following: (i) Overweight (body mass index, BMI ≥ 23 kg/m^2^) or obese (BMI ≥ 25 kg/m^2^) based on the criteria of the Asian population; (ii) Hypertension (blood pressure ≥ 130/85 mmHg or receiving antihypertensive drugs); (iii) Hyperglycemia (FBG ≥ 5.6 mmol/L or presence of type 2 diabetes mellitus); (iv) Hypertriglyceridemia (triglyceride ≥ 1.7 mmol/L) or low high-density lipoprotein cholesterol (HDL-C < 1.0 mmol/L for men and < 1.3 mmol/L for women) or receiving lipid-lowering drugs; (v) Hyperuricemia (uric acid > 7 mg/dL).^22^

### Histological assessment

Hematoxylin and Eosin (H&E) and Masson trichrome were available for all cases. Two experienced physicians evaluated the liver specimens according to the Fatty Liver Inhibition of Progression (FLIP) algorithm based on steatosis, activity, and Fibrosis (SAF) score and Metarvir scoring system blind to clinical profile. Steatosis (S) grade was evaluated by the percent of macrovesicular steatosis and mesovesicular steatosis hepatocytes: 0, < 5 %; 1, 5 %–33 %; 2, 33 %–66 %; 3, > 66 %. Ballooning hepatocytes were evaluated mainly by morphology: 0, none; 1, presence of clusters of rounds, pale cytoplasm hepatocytes with normal size; 2, similar to grade 1, but at least twice as large as normal hepatocytes. Lobular inflammation was classified into three grades: 0, none; 1, ≤ 2 foci per 20 × ; 2, > 2 foci per 20 × . Fibrosis (F) was still graded based on the NASH Clinical Research Network (NASH-CRN) staging system. Interface hepatitis was divided into four grades: 1, None of interface hepatitis; 2, Mild interface hepatitis; 3, Moderate interface hepatitis; 4, Severe interface hepatitis. NAFLD was diagnosed as the presence of greater than 5 % steatosis, and NASH was the presence of the above 3 histological features (steatosis, ballooning, and lobular inflammation).^23^ Significant Fibrosis (SF) and Advanced Fibrosis (AF) were considered as F ≥ 2 and F ≥ 3, respectively.

### Clinical endpoint

The clinical endpoint was virological response, defined as undetectable serum HBV DNA below the lower limit of detection (<500 IU/mL) at 48 weeks of antiviral monotherapy.

### Follow-up surveillance

During the follow-up period, patients were required to assess biochemical indicators and virological indicators approximately every 3 months at the studied center. Treatment adherence was assessed by patients' self-reports (asking if the patient took the medication on time) and medication dispensing records (checking medication dispensing records to ascertain whether patients prescribed medicine regularly). In the present study, all patients had good treatment adherence and were able to take drugs daily.

### Alcohol consumption

Given the influence of alcohol on antiviral efficacy, the authors collected the history of alcohol consumption of patients. Of the 120 patients enrolled in the analysis, 110 (91.7 %) cases had no history of alcohol consumption, 3 (2.5 %) cases had abstained from alcohol more than 6 months, 3 (2.5 %) cases were infrequent drinkers, and 4 (3.3 %) cases (all males) consumed alcohol less than one standard-drink before the initiation of antiviral therapy. Infrequent drinker is defined as those who consumed 1‒11 standard drinks in the past year. One standard drink is defined as one that contains 10 g of pure alcohol according to World Health Organization (WHO) criteria.^24^

However it should be emphasized that physicians would advise patients to quit drinking during antiviral therapy and ask whether patients drink alcohol at each follow-up visit. All patients were able to comply with the medical advice at the studied center. Hence, the effect of alcohol consumption on antiviral efficacy was not considered in the present study.

### Statistical analysis

All data was analyzed by R software (version 4.2.1). Quantitative variables were described as means ± Standard Deviation (SD) or medians (Q1, Q3). Categorical and ordinal variables were expressed as percentages. Comparisons were using Kruskal-Wallis test Chi-Square test, or Fisher's exact test. Factors found to be associated with concurrent NAFLD on univariate logistic regression at a probability threshold of <0.10 were included in multivariate logistic regression. The antiviral response was estimated by the Kaplan-Meier method and compared by a log-rank test. A Cox Proportional Hazards (PH) regression model was established to determine whether clinical of factors were independently associated with antiviral response. As the curves overlapped or even intersected before 8 weeks of follow-up when investigating the effect of histological characteristics, the authors adopted the eighth week as the cut-off value for the study, and mainly focused on the effect of factors on the efficacy after 8 weeks of follow-up. The PH assumption was tested by the Schoenfeld residual test, and p > 0.05 showed that the PH assumption was met (Figs. S2‒3). P-value < 0.05 was considered as statistically significant.

## Results

### Baseline characteristics

A total of 120 treatment-naïve CHB patients who underwent liver biopsy were enrolled in this study. The median age was 40.3 years old. Table 1, Table 2 show the clinical and pathological characteristics of patients. Compared with patients without NAFLD, those with NAFLD were primarily male (81.4 %) and more likely to be overweight/obese, hypertension, hypertriglyceridemia, hyperuricemia, and higher γ-GT (p < 0.05). The entire cohort had a median viral load of 6 log_10_ IU/mL and 58.3 % of those were HBeAg-positive. There were no differences (p > 0.05) among the three groups (CHB vs. CHB+NAFL vs. CHB+NASH) for viral load, HBeAg positive rate, ALT levels, and antiviral drug type.

**Table 1:** Clinical characteristics of patients and antiviral response rate.

Parameters	ALL (n = 120)	CHB (n = 61)	CHB+NAFLD (n = 59)	CHB+NAFL (n = 22)	CHB+NASH (n = 37)	p*
Age (years)	40.3 ± 10.9	39.0 ± 11.1	41.7 ± 10.4	38.7 ± 9.5	43.5 ± 10.7	0.102
Gender, male (%)	76 (63.3)	28 (45.9)^b^^,^^c^	48 (81.4)	19 (86.4)^a^	29 (78.4)^a^	<0.001
Body mass index (kg/m^2^)	23.6±3.2	22.0±3.0^b^^,^^c^	25.3±2.6	24.6±2.2^a^	25.7±2.8^a^	<0.001
Normal (%)	48 (40.0)	39 (63.9)	9 (15.3)	5 (22.7)	4 (10.8)	<0.001
Overweight (%)	35 (29.2)	14 (23.0)	21 (35.6)	7 (31.8)	14 (37.8)	
Obese (%)	37 (30.8)	8 (13.1)	29 (49.2)	10 (45.5)	19 (51.4)	
Hypertension (%)	12 (10.0)	4 (6.6)^c^	8 (13.6)	0 (0)^c^	8 (21.6)^a^^,^^b^	0.014
Hyperglycemia (%)	20 (16.7)	8 (13.1)	12 (20.3)	5 (22.7)	7 (18.9)	0.464
Hypertriglyceridemia (%)	17 (14.2)	3 (4.9)^c^	14 (23.7)	3 (13.6)	11 (29.7)^a^	0.003
Low HDL-C (%)	33 (27.5)	14 (23.0)	19 (32.2)	7 (31.8)	12 (32.4)	0.525
Hyperuricemia (%)	20 (16.7)	3 (4.9)^c^	17 (28.8)	4 (18.2)	13 (35.1)^a^	<0.001
MRR (%)	96 (80.0)	42 (68.9)^b^^,^^c^	54 (91.5)	19 (86.4)^a^	35 (94.6)^a^	0.006
Total bilirubin (µmol/L)	14.6 (10.7, 19.7)	14.0 (9.9, 21.0)	15.0 (11.0, 19.5)	16.7 (14.1, 19.9)	14.3 (10.4, 19.3)	0.366
Albumin (g/L)	44.2 (40.8, 47.9)	43.1 (39.4, 46.7)^c^	45.2 (42.4, 48.7)	44.3 (42.4, 48.5)	46.2 (42.4, 48.8)^a^	0.031
Globulin (g/L)	27.5 ± 4.4	27.9 ± 4.9^b^	27.1 ± 3.8	25.0 ± 2.9^a^^,^^c^	28.3 ± 3.8^b^	0.008
ALT (U/L)	44.1 (24.9, 82.1)	41.2 (22.0, 67.2)	50.5 (28.9, 99.9)	44.0 (20.7, 84.1)	52.6 (31.1, 105.2)	0.111
AST (U/L)	33.5 (24.0, 55.5)	32.8 (24.2, 60.5)	34.2 (22.0, 49.7)	28.6 (19.3, 46.1)	35.8 (26.3, 52.7)	0.456
γ-GT (U/L)	31.5 (18.1, 51.0)	26.0 (14.0, 51.5)^c^	33.4 (25.6, 51.0)	26.9 (18.0, 37,2)	39.9 (29.6, 56.1)^a^	0.028
ALP (U/L)	72.4 (62.3, 85.9)	70.0 (60.0, 85.3)	76.0 (64.8, 86.3)	73.3 (60.8, 90.9)	76.0 (68.0, 85.9)	0.256
FBG (mmol/L)	4.9 (4.6, 5.5)	4.8 (4.4, 5.2)	5.0 (4.7, 5.7)	4.9 (4.4, 5.8)	5.0 (4.8, 5.5)	0.094
HBV-DNA (log_10_ IU/mL)	6.0 (4.7, 7.9)	6.2 (5.0, 7.9)	6.0 (4.3, 8.0)	5.4 (4.2, 7.4)	6.1 (4.2, 8.1)	0.608
LVL (< 6 log_10_ IU/mL)	59 (49.2)	30 (49.2)	29 (49.2)	13 (59.1)	16 (43.2)	0.500
HVL (≥ 6 log_10_ IU/mL)	61 (50.8)	31 (50.8)	30 (50.8)	9 (40.9)	21 (56.8)	
HBeAg positive (%)	70 (58.3)	39 (63.9)	31 (52.5)	12 (54.5)	19 (51.4)	0.466
NAs type (%)						0.267
ETV	66 (55.0)	30 (49.2)	36 (61.0)	14 (63.6)	22 (59.5)	
TDF	22 (18.3)	11 (18.0)	11 (18.6)	6 (27.3)	5 (13.5)	
TAF	24 (20.0)	14 (23.0)	10 (16.9)	1 (4.5)	9 (24.3)	
TMF	8 (6.7)	6 (9.8)	2 (3.4)	1 (4.5)	1 (2.7)	
Virological response (%)	101 (84.2)	52 (85.2)	49 (83.1)	20 (90.9)	29 (78.4)	0.419

Data are presented as number (%) or mean ± SD or median (Q1, Q3).

∗p-value for comparison among 3 groups of CHB, CHB+NAFL and CHB+NASH.

a p < 0.05 versus CHB group.

b p < 0.05 versus CHB+NAFL group.

c p < 0.05 versus CHB+NASH group. ALP, Alkaline Phosphatase; ALT, Alanine Aminotransferase; AST, Aspartate Aminotransferase; CHB, Chronic Hepatitis; ETV, Entecavir; FBG, Fasting Blood Glucose; γ-GT, γ-Glutamyl Transferase; HBeAg, Hepatitis B e Antigen; HBV-DNA, Hepatitis B Virus Deoxyribonucleic Acid; HVL, High Viral Load; LVL, Low Viral Load; MRR, Metabolic-Related Risk; NAFL, Non-Alcoholic Fatty Liver; NAFLD, Non-Alcoholic Fatty Liver Disease; NAs, Nucleos(t)ide Analogues; NASH, Non-Alcoholic Steatohepatitis; TAF, Tenofovir Alafenamide Fumarate; TDF, Tenofovir Disoproxil Fumarate; TMF, Tenofovir Amibufenamide.

**Table 2:** Histological characteristics of patients.

Parameters	ALL (n = 120)	CHB (n = 61)	CHB+NAFLD (n = 59)	CHB+NAFL (n = 22)	CHB+NASH (n = 37)	p^a^
Ballooning grade (%)						
0	62 (51.7)	40 (65.6)	22 (37.3)	22 (100)	0 (0)	
1	39 (32.5)	11 (18.0)	28 (47.5)	0 (0)	28 (75.7)	
2	19 (15.8)	10 (16.4)	9 (15.3)	0 (0)	9 (24.3)	
Lobular inflammation (%)						
0	6 (5.0)	6 (9.8)	0 (0)	0 (0)	0 (0)	
1	49 (40.8)	24 (39.3)	25 (42.4)	15 (68.2)	10 (27.0)	
2	65 (54.2)	31 (50.8)	34 (57.6)	7 (31.8)	27 (73.0)	
Interface hepatitis (%)						0.687
1	29 (24.2)	17 (27.9)	12 (20.3)	4 (18.2)	8 (21.6)	
2	65 (54.2)	28 (45.9)	37 (62.7)	17 (77.3)	20 (54.1)	
3	24 (20.0)	14 (23.0)	10 (16.9)	1 (4.5)	9 (24.3)	
4	2 (1.7)	2 (3.3)	0 (0)	0 (0)	0 (0)	
Fibrosis stage (%)						0.407
0	6 (5.0)	0 (0)	6 (10.2)	2 (9.1)	4 (10.8)	
1	47 (39.2)	26 (42.6)	21 (35.6)	9 (40.6)	12 (32.4)	
2	42 (35.0)	20 (32.8)	22 (37.3)	9 (40.9)	13 (35.1)	
3	18 (15.0)	12 (19.7)	6 (10.2)	2 (9.1)	4 (10.8)	
4	7 (5.8)	3 (4.9)	4 (6.8)	0 (0)	4 (10.8)	
Significant fibrosis (%)	66 (55.0)	35 (57.4)	32 (54.2)	11 (50.0)	21 (56.8)	0.855
Advanced fibrosis (%)	25 (20.8)	15 (24.6)	10 (16.9)	2 (9.1)	8 (21.6)	0.299

Data are presented as number (%).

a p-value for comparison among 3 groups of CHB, CHB+NAFL and CHB+NASH. CHB, Chronic Hepatitis; NAFL, Non-Alcoholic Fatty Liver; NAFLD, Non-Alcoholic Fatty Liver Disease; NASH, Non-Alcoholic Steatohepatitis.

As for histological features, CHB patients with NASH had more severe lobular inflammation and hepatocyte ballooning degeneration compared to other groups. However, interface hepatitis and fibrosis grade were not different among the three groups (p > 0.05).

### Factors associated with concomitant NAFLD in CHB patients

The incidence of concomitants with NAFLD and NASH among CHB patients was 49.2 % and 30.8 %, respectively. The factors associated with the presence of NAFLD are presented in Table 3. In the univariable analysis, male (Odd Ratio [OR = 5.143], 95 % Confidence interval [95 % CI: 2.251‒11.751], p < 0.001), overweight (OR = 9.848, 95 % CI: 4.079‒23.776, p < 0.001), hypertriglyceridemia (OR = 6.015, 95 % CI: 1.629‒22.210, p = 0.007), hyperuricemia (OR = 7.825, 95 % CI: 2.154‒28.429, p = 0.002), metabolic-related risk (OR = 4.886, 95 % CI: 1.685‒14.165, p = 0.003), albumin (OR = 1.105, 95 % CI: 1.024‒1.191, p = 0.010), and FBG (OR = 1.754, 95 % CI: 1.042‒2.954, p = 0.034) were significantly associated with concomitant NAFLD. Further multivariate analyses revealed that male (OR = 4.222, 95 % CI: 1.620‒11.003, p = 0.003) and overweight (OR = 8.709, 95 % CI: 3.355–22.606, p < 0.001) were correlated with concomitant NAFLD. There was no association between virological factors and concurrent NAFLD.

**Table 3:** Factors associated with NAFLD in CHB patients.

Factors	Univariate analysis		Multivariate analysis	
	OR (95% CI)	p	OR (95% CI)	p
Age (years)	1.024 (0.990, 1,059)	0.173		
Gender, male (%)	5.143 (2.251, 11.751)	<0.001	4.222 (1.620, 11.003)	0.003
BMI ≥ 23 kg/m^2^ (%)	9.848 (4.079, 23.776)	<0.001	8.709 (3.355, 22.606)	<0.001
Hypertension (%)	2.235 (0.635, 7.867)	0.210		
Hyperglycemia (%)	1.691 (0.637, 4.493)	0.292		
Hypertriglyceridemia (%)	6.015 (1.629, 22.210)	0.007		
Low HDL-C (%)	1.595 (0.710,3.581)	0.258		
Hyperuricemia (%)	7.825 (2.154, 28.429)	0.002		
Metabolic-related risk (%)	4.886 (1.685, 14.165)	0.003		
Total bilirubin (µmol/L)	0.989 (0.969, 1010)	0.317		
Albumin (g/L)	1.105 (1.024, 1.191)	0.010		
ALT (U/L)	0.999 (0.997, 1.001)	0.350		
AST (U/L)	0.994 (0.988, 1.001)	0.105		
GGT (U/L)	1.000 (0.995, 1.005)	0.964		
ALP (U/L)	1.003 (0.987, 1.020)	0.686		
FBG (mmol/L)	1.754 (1.042, 2.954)	0.034		
HBV-DNA (log_10_ IU/mL)	0.928 (0.758, 1.136)	0.467		
HBeAg positive (%)	0.625 (0.301, 1.297)	0.207		

ALT, Alanine Aminotransferase; AST, Aspartate Aminotransferase; GGT, Glutamyl Transferase; ALP, Alkaline Phosphatase; BMI, Body Mass Index; CI, Confidence Interval; FBG, Fasting Blood Glucose; HBeAg, Hepatitis B e Antigen; HBV-DNA, Hepatitis B Virus Deoxyribonucleic acid; NAFLD, Non-Alcoholic Fatty Liver Disease; OR, Odds Ratio.

### Impact of concurrent NAFL and NASH on antiviral response

**Fig. 2:**
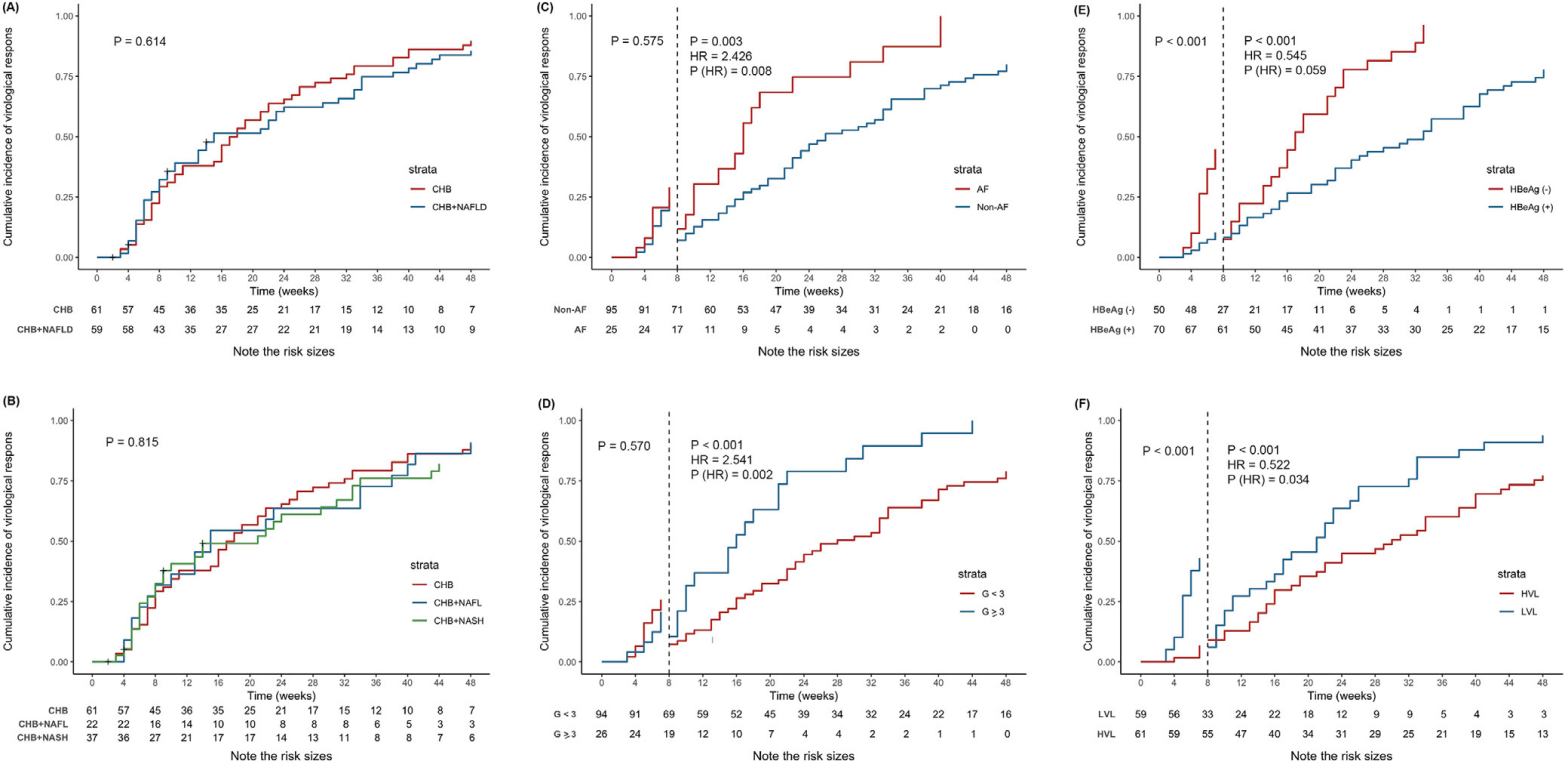
Kaplan-Meier estimated cumulative incidence of virological response at 48 weeks of follow-up. (A) Kaplan-Meier estimated cumulative incidence of virological response by the presence of NAFLD. (B) Kaplan-Meier estimated cumulative incidence of virological response by the presence of NAFL or NASH. (C) Kaplan-Meier estimated cumulative incidence of virological response by the presence of advanced fibrosis. Prior to 8-week of follow-up, advanced fibrosis had no impact on virological response, but after 8-week of follow-up, a 2.426-fold increased probability of virological response. (D) Kaplan-Meier estimated cumulative incidence of virological response by the presence of moderate-to-severe interface hepatitis. The result was similar with advanced fibrosis. After 8-week of follow-up, a 2.541-fold increased probability of virological response. (E) Kaplan-Meier estimated cumulative incidence of virological response by HBeAg status. After adjusting for viral load, advanced fibrosis, and interface hepatitis, HBeAg status no longer had impact on virological response. (F) Kaplan-Meier estimated cumulative incidence of virological response by HBV viral load, which was categorized into Low Viral Load (LVL, < 6 log_10_ IU/mL) and High Viral Load (HLV, ≥ 6 log_10_ IU/mL). And patients with LVL had a 1.916-fold (1/0.522-fold) increased probability of virological response compared with HVL after 8-week of follow-up. G < 3 meant no interface hepatitis or mild interface hepatitis; G ≥ 3 meant moderate-to-severe interface hepatitis. Abbreviations: CHB, chronic hepatitis B; CI, confidence interval; G, grade; HBeAg, hepatitis B e antigen; HR, hazard ratio; HVL, high viral load; LVL, low viral load; NAFL, non-alcoholic fatty liver; NAFLD, non-alcoholic fatty liver disease; NASH, non-alcoholic seatohepatitis.

During the 48-week follow-up period, 115 (95.8 %) patients completed the end of follow-up and 5 (4.2 %) patients were lost to follow-up. The overall cumulated incidence of virological response at the end of 48-week follow-up was 84.2 % and the median time to response was 17 weeks. The overall antiviral response was not different (p > 0.05 by log-rank test) among the three groups, with 48-week virological response rates of 85.2 %, 90.9 %, and 78.4 % in CHB, CHB+NAFL, and CHB+NASH groups, respectively (Fig. 2A‒B).

### Impact of viral and histological characteristics on antiviral response

Factors significantly correlated with virological response were estimated by Kaplan-Meier plots, and viral load, HBeAg status, advanced fibrosis, and moderate-to-severe interface hepatitis had impact on antiviral response (Fig. S1). In the multivariate cox regression model, high viral load (Hazard Ratio [HR = 0.522], 95 % CI: 0.286‒0.952, p = 0.034), advanced fibrosis (HR = 2.426, 95 % CI: 1.256‒4.686, p = 0.008), and moderate-to-severe interface hepatitis (HR = 2.541, 95 % CI: 1.406‒4.592, p = 0.002) had a significant impact on the antiviral response after 8-week of follow-up, while negative HBeAg was no longer associated with virological response when adjusted for viral load, advanced fibrosis, and interface hepatitis (Table 4). But viral load and HBeAg status still affected the virological response (p < 0.001) before 8-week of follow-up. Kaplan-Meier estimated cumulative incidence of virological response by the above four groups are shown in Fig. 2C‒F.

**Table 4:** Cox regression analysis after 8-week of follow-up.

Factors	Univariate analysis		Multivariate analysis	
	HR (95% CI)	p	HR (95% CI)	p
Viral load (%)	0.489 (0.297, 0.804)	0.005	0.522 (0.286, 0.952)	0.034
HBeAg status (%)	0.327 (0.191, 0.559)	<0.001	0.545 (0.290, 1.023)	0.059
Advanced fibrosis (%)	2.478 (1.351, 4.543)	0.003	2.426 (1.256, 4.686)	0.008
Interface hepatitis (%)	2.874 (1.624, 5.088)	<0.001	2.541 (1.406, 4.592)	0.002

CI, Confidence Interval; HBeAg, Hepatitis B e Antigen; HR, Hazard Ratio.

## Discussion

Concurrent NAFLD in chronic hepatitis B patients has an upward trend over time, with an overlapping prevalence of approximately 14 %‒67 % in the Asian population.^25^ In this study, the authors found that concurrent NAFLD and NASH were present in 49.2 % and 30.8 % of CHB patients, respectively. It is in line with that reported in previous studies. Surprisingly, nearly one-third of CHB patients at the studied center had comorbid NASH. Compared with simple hepatic steatosis, NASH portended a more rapid progression to cirrhosis and clinical outcomes.^26^ Therefore, it is plausible to speculate that CHB concomitant with NASH can accelerate the progression of disease. Mounting evidence has supported this viewpoint. A retrospective study reported that NASH was the independent risk factor of significant fibrosis (HR = 10.00, 95 % CI: 2.08‒48.50) and advanced fibrosis (HR = 3.45, 95 % CI: 1.11‒10.70) rather than simple hepatic steatosis.^10^ Another two prospective studies have also shown that severe hepatic steatosis was associated with fibrosis progression among CHB patients.^27^^,^^28^ Mak et al.^3^ reported similar findings, with a 2-fold increased hazard of fibrosis progression due to severe steatosis at a 3-year follow-up. However, this result has not been demonstrated in the present study. Neither hepatic steatosis nor NASH seemed to be related to exacerbating fibrosis. Differences in inclusion criteria of the study population (treatment-naïve CHB patients), sample size, and measures (liver biopsy or VCTE) for defining fibrosis and steatosis might explain the inconsistency among these studies.

Similar to other studies, the authors found that males and overweight were predictors for concurrent NAFLD in CHB patients, which were also risk factors in the general population.^9^^,^^29^, ^30^, ^31^, ^32^ Although FBG was not a risk factor of concurrent NAFLD in the multivariate analysis, it indeed elevated in CHB patients with concomitant NAFLD. This might suggest that the mechanism of insulin resistance promoted fatty acid accumulation in hepatocytes leading to liver injury and inflammation. Experimental animal studies indicated that HBV-related molecules might be associated with the primary pathway involved in the occurrence of hepatic steatosis,^33^ but virological indicators (including HBV load and HBeAg status) were not linked to the presence of NAFLD at the studied center. Other studies also failed to confirm the relationship between virological characteristics and the existence of NAFLD.^10^^,^^34^ Furthermore, Seto et al.^27^ showed that decreased BMI (OR = 0.68, 95 % CI: 0.48‒0.97) could contribute to fibrosis or cirrhosis regression. Overall, it appeared that host metabolic factors and gender rather than HBV characteristics were responsible for concurrent NAFLD among CHB patients, highlighting that antiviral therapy accompanied by modification of metabolic disorders was the key to controlling disease progression among CHB patients concomitant with NAFLD.

NAs are widely used for the treatment of CHB and for the prevention of disease progression and liver-related adverse outcomes in clinical practice. As concurrent NAFLD was previously reported to be capable of inhibiting HBV replication, it should be explored whether concurrent NAFLD affects antiviral therapy response. However, contradictory conclusions were found in this regard.^13^, ^14^, ^15^, ^16^, ^17^, ^18^, ^19^^,^^34^ Chen et al.^14^ reported that ALT normalization and HBV-DNA clearance rates were worse in high CAP patients compared to that in normal CAP patients at 12, 24, and 48-weeks. A meta-analysis showed that the virological (66.2 % vs. 72.3 %, p = 0.006) and biochemical (62.7 % vs. 75.8 %, p = 0.002) responses at 48 and 96 weeks were significantly lower in CHB patients with hepatic steatosis diagnosed by ultrasound when compared to those without.^15^ However, some studies demonstrated that concurrent NAFLD had no impact on antiviral therapy. A retrospective study including 555 CHB patients found that concurrent NAFLD (assessed by ultrasound or computerized tomography or magnetic resonance imaging) was not associated with complete virologic suppression (86 % vs. 88 %, p = 0.550) or biochemical response (38 % vs. 41 %, p = 0.320) during a 60-month follow-up.^19^ Similar results were found in three other studies in CHB patients with NAs treatment.^13^^,^^17^^,^^35^

Given that previous clinical studies focused on the impact of concurrent simple steatosis on antiviral therapy, limited studies investigated the impact of concurrent NASH. The authors speculated that disease subtypes (NAFL or NASH) and different diagnosis methods, such as abdominal ultrasound, computerized tomography, magnetic resonance imaging, and VCTE, might led to this phenomenon. Therefore, the authors analyzed the effect of concurrent liver biopsy-proven NAFLD on antiviral treatment at the studied center and found that virological response rates and response status during the 48-week follow-up were not significantly different among CHB, CHB+NAFL, and CHB+NASH groups, which demonstrated that NAFL or NASH had no influence on antiviral efficacy (all p > 0.05).

In order to determine the potential factors that affected virological response, the authors analyzed demographic data (age and gender), metabolic-related risk, aminotransferase, and histological characteristics and found that predictors for virological response were baseline low viral load, negative HBeAg status, advanced fibrosis, and moderate-to-severe interface hepatitis in the univariate cox regression model after 8-week of follow-up. However, HBeAg status was not different in the multivariate cox regression model (p > 0.05). It might be related to a positive correlation between viral load and HBeAg status (*r*_s_ = 0.589, p < 0.001) (Table S1). According to the histological injury pattern of CHB, moderate-to-severe interface hepatitis suggested an active stage of disease and might respond better to antiviral therapy.^36^ Additionally, the authors also found that almost half of the advanced fibrosis patients had moderate-to-severe interface hepatitis (while only 14.7 % of non-advanced fibrosis patients had moderate-to-severe-interface hepatitis) at the studied center, which might explain the better response in advanced fibrosis patients.

Notably, it was obviously observed that the cumulative curves overlapped and even intersected before 8-week of follow-up in the advanced fibrosis and moderate-to-severe interface hepatitis groups (Fig. S1G, H), so the authors took the eighth week as a cut-off value for the segmental study and mainly investigated the effect of factors on the virological response after 8-weeks of follow-up.

There were also limitations in the present study. Firstly, although no difference was found among the three groups for oral antiviral drug types, it was better to use the same one. Besides, the number of patients was unevenly distributed among the three groups. A large-scale, inter-group balanced study was needed to verify the present findings. Finally, due to the retrospective study, the lack of anthropometric measurements such as waist and hip circumference prevented the validation of the association of metabolic syndrome with concurrent NAFLD.

## Conclusion

This study highlights that concurrent hepatic steatosis and even non-alcoholic steatohepatitis are common in CHB patients, implying the importance of screening for NAFLD in the management of the CHB population. Furthermore, the present findings demonstrated the presence of NAFL or NASH had no impact on the antiviral response, whereas viral load, the degree of fibrosis, and interface hepatitis affected antiviral efficacy, especially in patients with low viral load (< 6 log_10_ IU/mL), advanced fibrosis, and moderate-to-severe interface hepatitis, leading to a good virological response.

## Funding

This work was supported by the National Natural Science Foundation (No. 81970454), the Key Projects of Jiangsu Provincial Health Commission (No. ZD2021061), the Postgraduate Research & Practice Innovation Program of Jiangsu Province (No. KYCX23_2102), Jiangsu Province Traditional Chinese Medicine Science and Technology Development Program (No. YB2020037), and Natural Science Foundation of Nanjing University of Chinese Medicine (No. XZR2020071).

## Ethics approval and consent to participate

This study conformed to the ethical guidelines of the Declaration of Helsinki and was approved by the Ethics Committee of the Second Hospital of Nanjing (No. 2022-LY-kt101). The data were anonymous, and the requirement for informed consent was waived by the Medical Ethics Committee of the Second Hospital of Nanjing.

## Availability of data

All data will be available from the corresponding author upon request.

## CRediT authorship contribution statement

**Miao-Yang Chen:** Conceptualization, Methodology, Data curation, Formal analysis, Writing – original draft, Writing – review & editing. **Shun-Xin Li:** Data curation, Formal analysis, Writing – original draft, Writing – review & editing. **Zhi-Xiang Du:** Data curation, Formal analysis, Writing – review & editing. **Qing-Fang Xiong:** Conceptualization, Methodology, Writing – review & editing. **Yan-Dan Zhong:** Data curation, Formal analysis, Writing – review & editing. **Du-Xian Liu:** Data curation, Formal analysis, Writing – review & editing. **Yong-Feng Yang:** Writing – review & editing.

## Declaration of competing interest

The authors declare no conflicts of interest.

## Appendix Supplementary materials

Image, application 1
