# Supplementary material for: Liver biopsy-proven non-alcoholic fatty liver disease predicts no impact on antiviral response in patients with chronic hepatitis B
Source: Clinics (Sao Paulo). 2024 Sep 26;79:100493. doi: 10.1016/j.clinsp.2024.100493 (PMC11467630; doi:10.1016/j.clinsp.2024.100493)
Supplement: Supplementary file 1 [file mmc1.docx]

**CLINICS-D-23-00668_Supplementary Material**

**Table S1** Spearman correlation analysis.

| **Variables** | **Spearman** | **p** |
| --- | --- | --- |
| Viral load |  |  |
| HBeAg status | 0.589 | <0.001 |
| Advanced fibrosis | 0.094 | 0.307 |
| Moderate-to-severe interface hepatitis | 0.113 | 0.221 |

HBeAg, Hepatitis B e Antigen.

**Figure S1** Factors associated with virological response estimated by Kaplan-Meier plots. G <3 meant no interface hepatitis or mild interface hepatitis; G ≥3 meant moderate-to-severe interface hepatitis. Abbreviations: AF, Advanced Fibrosis; ALT, Alanine Aminotransferase; CHB, Chronic Hepatitis B; G, Grade; HBeAg, Hepatitis B e Antigen; HVL, High Viral Load; LVL, Low Viral Load; MRR, Metabolic-Related Risk; NAFL, Non-Alcoholic Fatty Liver; NAFLD, Non-Alcoholic Fatty Liver Disease; NASH, Non-Alcoholic Steatohepatitis.


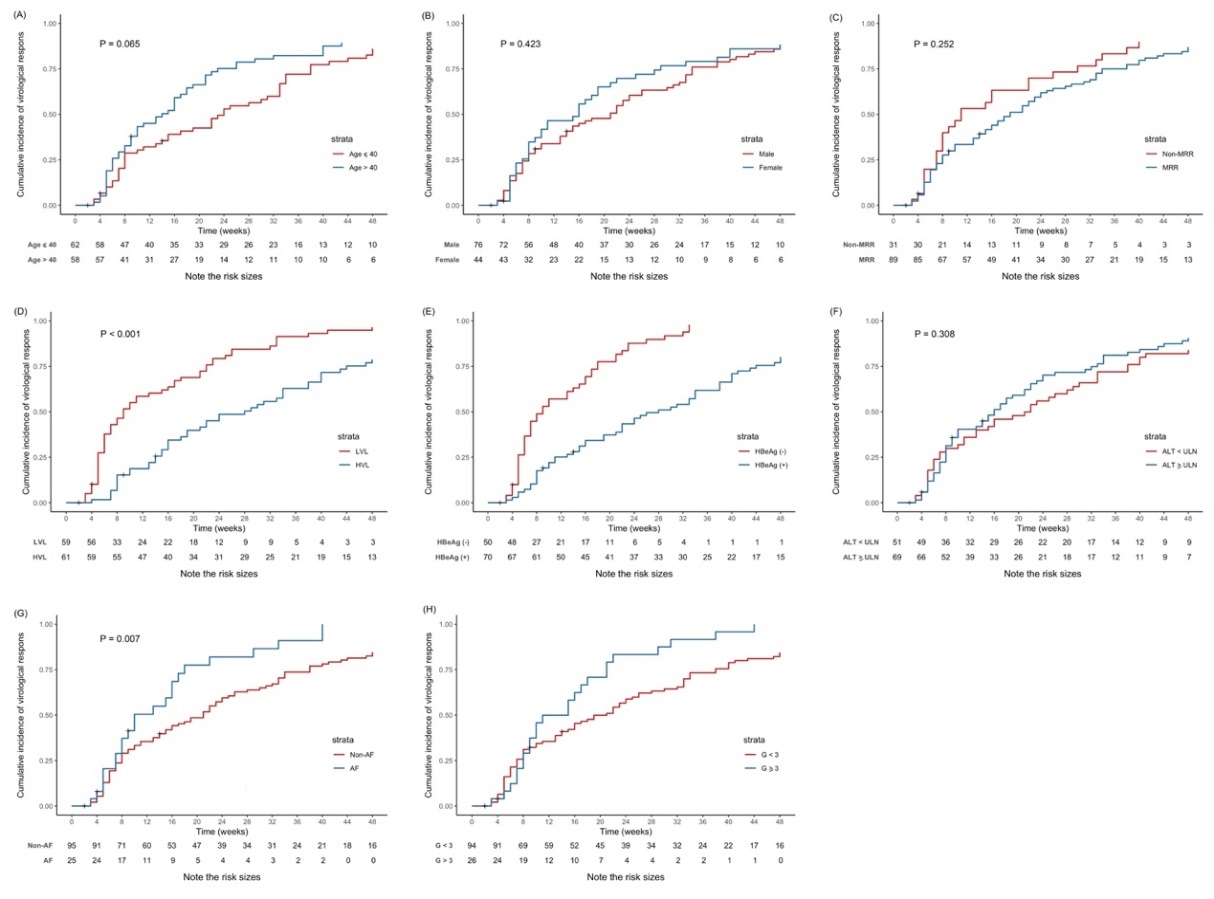


**Figure S2** Proportional Hazards (PH) assumption of univariate cox regression tested by Schoenfeld residual test; p > 0.05 shows that the PH assumption is met. Abbreviations: HBeAg, Hepatitis B e Antigen.


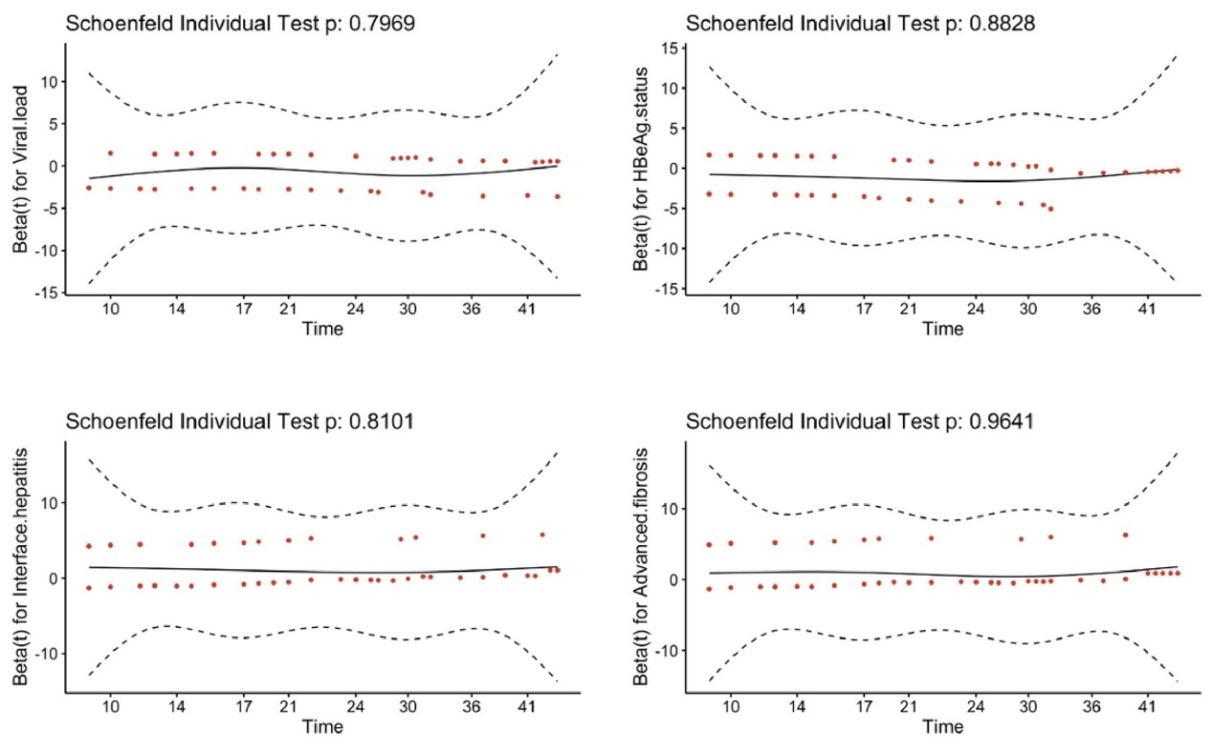


**Figure S3** Proportional Hazards (PH) assumption of multivariate cox regression tested by Schoenfeld residual test; p > 0.05 shows that the PH assumption is met. Abbreviations: HBeAg, Hepatitis B e Antigen.

_
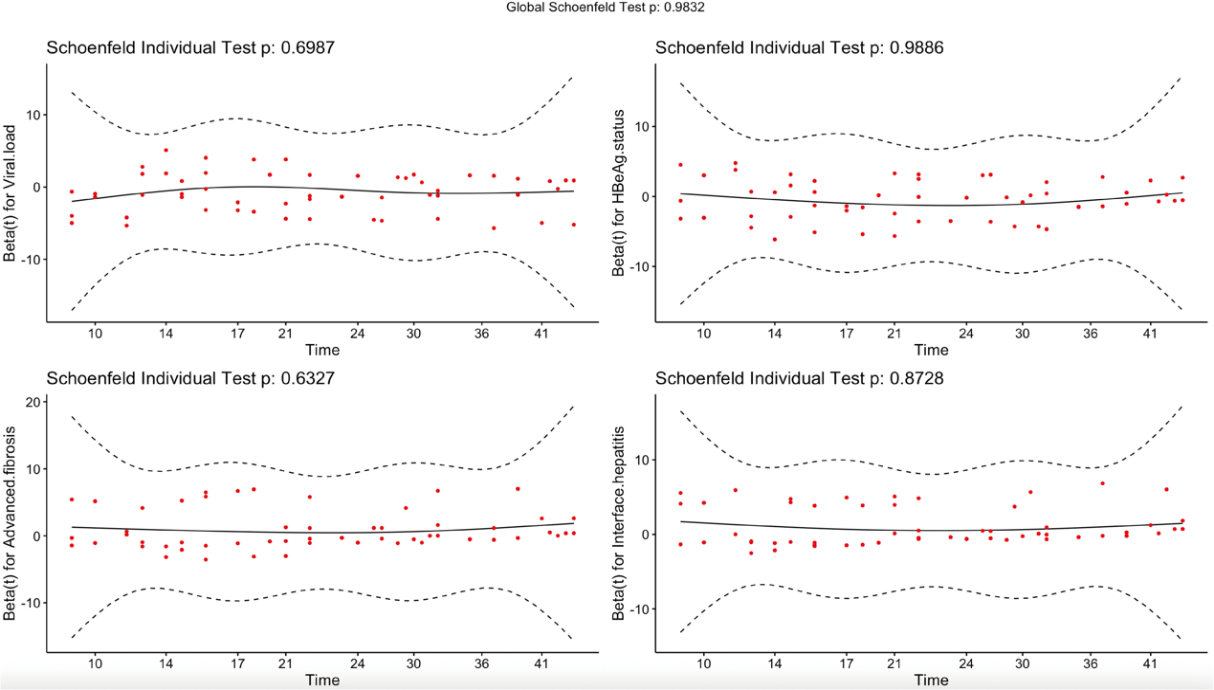
_
